# “Will you hear my voice?”: to engage older patients online, listen to them about their lives offline

**DOI:** 10.1186/s13584-020-00408-y

**Published:** 2020-10-06

**Authors:** Michael L. Millenson

**Affiliations:** Northwestern University Feinberg School of Medicine, Chicago, IL, USA and Health Quality Advisors LLC, Highland Park, IL USA

**Keywords:** Patient-centered care, Patient and family advisory councils (PFACs), COVID-19, Digital exclusion, Consumerism, HMOs, Patient portals, Online health services, Older adults, Patient engagement, Internet health

## Abstract

The scope of health information and health care services available online is rapidly expanding. At the same time, COVID-19 is causing vulnerable elders to reconsider in-person provider visits. In that context, recently published research by Y. Mizrachi et al. examining obstacles to the use of online health services (OHS) among adults age 50 and up takes on new importance. An iconic Israeli song begins, “Will you hear my voice?” (Hebrew Songs. Zemer Nugeh (Hatishmah Koli), 2020). What makes Mizrachi et al.’s findings particularly intriguing, despite several caveats, is the manner in which they demonstrated a commitment to genuinely listen to individual voices. The researchers spoke “openly and bluntly” with interviewees as peers and were rewarded with “specific, well-defined and applicable answers with the potential to be used.” The most striking findings came in candid answers that went beyond the factors intrinsic to the online offerings and addressed important factors in what regular Internet users often refer to as IRL (“in real life”), such as support from family. The necessity of avoiding preconceptions about the most effective manner to engage patients underscores the importance of patient and family advisory councils (PFACs). PFACs, increasingly being adopted by health care organizations globally, provide an ongoing ability to listen and respond to the “patient voice.” Effectively addressing obstacles to older adults’ use of the full range of online health resources will require the involvement not just of health plans and government, but also of voluntary organizations, providers, families and others integral to users’ offline “real lives.” Sustained, focused listening must be a central part of that effort.

The scope of health information and health care services available online is rapidly expanding. At the same time, COVID-19 is causing vulnerable elders to reconsider in-person provider visits. In that context, recent research by Y. Mizrachi et al. examining obstacles to the use of online health services (OHS) among adults age 50 and up [1] assumes new importance.

Using OHS to obtain exam results, make appointments and find medical information can be critically important to well-being. Unfortunately, as Seifert et al. recently noted, vulnerable populations such as older adults “tend to be excluded from digital services because they opt not to use the Internet, lack necessary devices and network connectivity or [through] inexperience using the technology.” Moreover, as a result of the pandemic they may “struggle with the double burden of social and digital exclusion” [2].

The in-depth interviews conducted by Mizrachi et al. offer clues to confronting this challenge. Their work comes with caveats: Interviews were conducted in mid-2014, making this the equivalent of a survey of iPhone 6 users just as the iPhone 12 is rolling out. Moreover, while the study makes a bow to Internet health information and telemedicine, it focuses on portal offerings by Israeli HMOs. Finally, interviewees aged 65 and over seem to constitute only about a third of respondents. Most of the interviewees were in the younger age-band of older adults.

Counter-balancing those limitations is that change in patient portals has been frustratingly slow; this somewhat-dated research is, alas, not outdated. Moreover, the obstacles identified in this age group were generally ones that even older populations would experience even more acutely. In addition, adding newer services such as Web-based videos and interactive education programs likely raises identified barriers even higher [3].

An iconic Israeli song begins, “Will you hear my voice?” [4] What makes Mizrachi et al.’s findings particularly intriguing is how they demonstrated a commitment to listening to individual voices, even while adhering to rigorous standards of interviewing and characterizing the information gathered. In contrast to the lip service often paid to the patient’s “holistic” needs, this research includes a genuine focus on how OHS fits into people’s lives.

Communication styles vary across cultures. Israeli society, for instance, is distinguished by the directness of its dialogue. From that perspective, the researchers noting that they spoke “openly and bluntly” with interviewees can be seen as treating them as peers. The reward for this approach (which may vary in other cultures) was “specific, well-defined and applicable answers with the potential to be used.”

Interviewee feedback on making portals more usable included complaints that the website font size was too small and the need to change passwords too frequent. These types of problems for older users are, of course, not confined to one country.

Disappointingly, one of the main explanations for non-usage was that interviewees were either unaware of the portal’s existence or unaware of its potential benefits. Conversely, the most encouraging reason for non-use was, “I am healthy.” If only that were the most common response!

The most striking findings came in candid answers going beyond factors specific to the online offerings and addressing the impact of “IRL” – what regular Internet users often refer to “in real life.” For instance, many interviewees who were not online acknowledged that in real life they had peers who used OHS, yet they themselves were” “stuck” in non-use because of a lack of motivation to change. Support from family – say, a helping hand from a grandson – was often critical in prompting non-users to give online a try.

Among the IRL suggestions offered by OHS users to expand and improve usage among peers were: more outreach to families, more “motivational” advertising and better training. Suggested changes online included more types of services and improved usability. The researchers noted that what some might seem to younger people to be minor technical glitches can be perceived by older people as “real and almost tangible barriers.”

Collectively, the voices of the middle-aged and elderly interviewees reflected frustration with a health care system that frequently seems deaf to their lived experience and uninterested in a genuine relationship. This is a familiar refrain that, again, is not unique to any one nation or even any one age group.

A recent analysis of the communication patterns of 300,000 diabetics sounded an important reminder about listening to individual needs. “Improving patient engagement cannot rely solely on technological solutions,” it concluded, but “must be accompanied by complementary means.” For instance, some patients may prefer text messages, while others prefer talking directly with a nurse or doctor [5]. At times, the most effective approach may be to integrate portal information into phone conversations or face-to-face contact [6].

The necessity of avoiding preconceptions about patient preferences underscores the importance of patient and family advisory councils (PFACs). PFACs, increasingly being adopted globally [7], provide an ongoing capability of listening and responding to the “patient voice.”

For instance, the U.S. health plan Kaiser Permanente has established member advisory councils with community members for operational feedback and “patient partner panels” for relevant research, such as examining patient portal use [8].

Low usage of patient portals has long been a problem [9], particularly among older adults [10]. Even as the variety and sophistication of online information and services expands – including telemedicine as a newly critical component of care – portals still play an important role in connecting patients with providers and payers.

In the pandemic era, addressing obstacles to older adults’ use of the full range of these online health resources has acquired new urgency. Doing so effectively, the research by Y. Mizrachi et al. shows, will require the involvement not just of health plans and government, but also of voluntary organizations, providers, families and others integral to users’ offline “real lives.”

Sustained, focused listening must be a central part of that effort. In a genuinely patient-centered environment, “Will you hear my voice?” should no longer linger as a plaintive and persistent patient question.
